# Reliability and validity of cross‑culturally adapted oral health‑related quality‑of‑Life instruments for Brazilian children and adolescents: a systematic review

**DOI:** 10.1186/s12903-024-03940-4

**Published:** 2024-02-10

**Authors:** Yure Gonçalves Gusmão, Frederico Santos Lages, José Cristiano Ramos Glória, Dhelfeson Willya Douglas-de-Oliveira

**Affiliations:** 1 Federal University of Jequitinhonha and Mucuri Valleys, Rua da Glória, 187, Centro, Diamantina, MG 39100-000 Brazil; 2 Federal University of Minas Gerais, Avenida Antônio Carlos, 6627, Belo Horizonte, MG 31270-901 Brazil

**Keywords:** Quality of life, Instruments, Cross-cultural adaptation, Psychometrics, Systematic review

## Abstract

**Objective:**

This systematic review aimed to review the reliability and validity of oral health-related quality of life (OHRQoL) questionnaires for Brazilian children and adolescents. Also, the cross-cultural adaptation was evaluated.

**Methods:**

This systematic review is registered in PROSPERO (CRD42022300018) and was performed based on the COSMIN guideline. Electronic searches were performed in the PubMed/MEDLINE, Web of Science, Lilacs, BVS (BIREME), Scielo, and Embase databases until March 2023 by two independent reviewers. There was no restriction on time or language. The following studies were included: validation studies and cross-cultural adaptation of OHRQoL instruments into Brazilian Portuguese; studies that evaluated the measurement properties of OHRQoL questionnaires in children and adolescents and that reported at least one of the measurement properties: reliability, internal consistency, error measurement, content validity, construct validity, criterion validity, discriminant validity, and/or convergent validity. The following were excluded: studies of systematic reviews of OHRQoL measures; studies reporting OHRQoL assessment through instruments; construction (development) and validation of a new instrument; questionnaires that had a single item; and validation for Portuguese from Portugal. The cross-cultural adaptation process and psychometrics of the included studies were verified.

**Results:**

6556 articles were identified, and 19 manuscripts were included. All studies were conducted in Brazil, and the age of the participants ranged from 2 to 15.42 years old. Sixteen articles presented the cross-cultural validation steps. Cronbach's alpha of the revised instruments ranged from 0.59 to 0.86.

**Conclusions:**

It can be concluded that most studies provided information and evidence regarding validity, reliability, translation, and cultural adaptation.

**Supplementary Information:**

The online version contains supplementary material available at 10.1186/s12903-024-03940-4.

## Introduction

Quality of life is intertwined with an individual's perception within their cultural and value systems, aligned with their goals, expectations, standards, and perspectives [1, 2]. Based on this premise, measures of health-related quality of life have been developed, known as patient-reported outcome measures. These measures aim to gauge the impact of a health condition or treatment from the patient's psychosocial viewpoint, contrasting with the professional approach [3].

The evaluation of oral health based solely on clinical criteria falls short of measuring the genuine impact of oral issues on people's lives [4]. Consequently, to comprehensively understand the effects of changes in oral health assessment methods, the development of oral health-related quality of life (OHRQoL) questionnaires has been encouraged, and increasingly utilized in research [5]. Nonetheless, some of these instruments have limitations in their applicability, given that most are developed in English and countries with social and cultural realities distinct from Brazil [6]. Hence, these questionnaires must undergo cross-cultural adaptation and psychometric validation before implementation in Brazil [7].

Standardized guidelines for this validation and cross-cultural adaptation outline a process comprising stages aimed at ensuring equivalence and maintaining quality [6]. Moreover, these instruments must substantiate the accuracy of their results through psychometric properties, serving as benchmarks for measurement quality. These criteria encompass content validity, internal consistency, construct validity, responsiveness, reliability, reproducibility, convergent validity, discriminant validity, and interpretation [7, 8].

These cross-culturally adapted questionnaires, translated into Brazilian Portuguese and deemed suitable for use, have facilitated the assessment of how oral health impacts quality of life [9]. Notably, most of these questionnaires target adults, posing a significant challenge in evaluating oral health-related quality of life in children [10, 11]. Given the multitude of pediatric oral disorders with potential negative impacts on quality of life, there's a need for measures documenting oral health outcomes in these younger populations [12].

However, to circumvent reliability issues linked to cross-cultural adaptations, a critical evaluation of these translated versions is necessary to verify their adapted measures and preservation of psychometric properties.

This study aimed to review the reliability and validity of adapted OHRQoL questionnaires for children and adolescents, assessing their suitability for research and clinical practice in Brazil. Additionally, it critically evaluated and summarized the cross-cultural adaptation process of the revised questionnaires.

## Methodology

The present systematic review is registered in PROSPERO (CRD42022300018) and was performed based on the COSMIN guideline for systematic reviews of patient-reported outcome measures (https://www.cosmin.nl/).

### Focus question

The COSMIN manual was used to establish the study question and to conduct the search. According to the manual, the question should include the following four key elements: 1) the construct; 2) the population(s); 3) the type of instrument(s); and 4) the measurement properties of interest. Hence, the focus question became:

What is the reliability and validity of transculturally adapted and translated questionnaires used to assess OHRQoL in Brazilian children and adolescents?

### Eligibility criteria

For this systematic review, studies were included based on the following criteria: validation studies and cross-cultural adaptation of OHRQoL instruments into Brazilian Portuguese, studies evaluating measurement properties of OHRQoL questionnaires in children/adolescents, and those reporting at least one of these measurement properties: reliability, internal consistency, measurement error, content validity, construct validity, criterion validity, discriminant validity, and/or convergent validity. Excluded from consideration were systematic reviews of OHRQoL measures, studies solely reporting OHRQoL assessment through instruments, the development and validation of new instruments, questionnaires consisting of a single item, and validations conducted specifically for Portuguese from Portugal.

### Search strategy

The studies were acquired through electronic searches conducted in the PubMed/MEDLINE, Web of Science, Lilacs, VHL (BIREME), Scielo, and Embase databases. Keywords were utilized and searched within Health Sciences Descriptors (DeCs), Medical Subject Headings (MeSH), and published manuscripts focusing on oral health-related quality of life. The boolean operators AND and OR were employed alongside the following terms: quality of life, oral health quality of life, instrument, scale, questionnaire, measurement, measurement tool, psychometrics, reliability, validity, instrument validation, cross-cultural adaptation, instrument translation, Brazilian version, Brazil, Portuguese, Brazilian Portuguese. A generic search strategy was tailored to suit the specific attributes of each database, aiming to identify relevant studies for this review (Table 1). Articles and abstracts from databases were sought without language or time restrictions. Furthermore, an additional search was conducted for grey literature using Google Scholar. All included study references were reviewed to identify supplementary studies. Searches in these databases were conducted until March/2023.

**Table 1:** Search strategy adapted for each database

Database	Search query
PUBMED/MEDLINE LILACS VHL (BIREME)	(quality of life OR oral health quality of life OR instrument OR scale OR questionnaire OR measurement OR measurement tool) AND (psychometrics OR reliability OR validity) AND (instrument validation OR cross cultural adaptation OR instrument translation) AND (brazilian version OR Brazil OR Portuguese OR Brazilian Portuguese)
Web of Science	**#1:** TS=(quality of life OR oral health quality of life OR instrument OR scale OR questionnaire OR measurement OR measurement tool)
	**#2:** TS=(psychometrics OR reliability OR validity)
	**#3:** TS=(instrument validation OR cross cultural adaptation OR instrument translation)
	**#4:** TS=(brazilian version OR Brazil OR Portuguese OR Brazilian Portuguese)
	**#5:** #1 AND #2 AND #3 AND #4
Scielo	(quality of life OR oral health quality of life OR instrument OR scale OR questionnaire OR measurement OR measurement tool) AND (psychometrics OR reliability OR validity)
Embase	**#1:** (quality of life OR oral health quality of life OR instrument OR scale OR questionnaire OR measurement OR measurement tool)
	**#2:** (psychometrics OR reliability OR validity)
	**#3:** (instrument validation OR cross cultural adaptation OR instrument translation)
	**#4:** (brazilian version OR Brazil OR Portuguese OR Brazilian Portuguese)
	**#5:** #1 AND #2 AND #3 AND #4

### Studies selection

The Rayyan tool (https://rayyan.qcri.org/welcome) was used in the selection of studies, duplicates identification, management, and citation of references during the development of this review [13]. The study selection process was performed by three reviewers (DWDdeO, FSL, and YGG) in two phases. In the first phase, reviewers independently identified all relevant studies through electronic search methods based on inclusion criteria applied to titles and abstracts. The full text was pre-selected for studies that appeared to meet the inclusion criteria or for which insufficient data were found in the title and abstract to make a clear decision. In the second phase, the pre-selected studies were read in full by the same researchers to define whether the study met the inclusion criteria. When necessary, the authors of the studies were contacted by email to clarify questions related to the research. Studies excluded at this or subsequent stages were recorded, along with the reasons for rejection. Observational studies that met the eligibility criteria were included in the final analysis and submitted to data synthesis. Articles identified twice or more were considered only once. Disagreements were resolved by consensus among the three reviewers. This procedure was applied at all stages. The reviewers were trained for database use before the study.

### Data extraction

The data were qualitatively recorded to allow comparisons of the selected studies. Each researcher qualitatively evaluated the studies. Data were collected on the following items: author, year of publication, country, study design, characteristics of the participants (gender and mean age), original language of the instrument, cross-cultural adaptation process, target population, main reported results, conclusion, name of the questionnaire, acronym, generality or specificity of the instrument, method of conclusion, domains, number of items, score, period of evaluation, time of completion, availability of the questionnaire in Brazilian Portuguese, Cronbach’s alpha, internal consistency, criterion validity, construct validity, reliability, discriminant validity, general ICC value, translation, back-translation, synthesis, committee approach, pre-test and psychometric evaluation.

### Measurement properties assessment

The psychometric properties of oral health-related quality of life questionnaires identified were then evaluated according to nine criteria: content validity, internal consistency, criterion validity, construct validity, reproducibility, responsiveness, floor, ceiling effects, and interpretability. Each scale received a positive (+), undetermined (?), or negative (-) rating for each of these measures, or a rating of 0 if no information is available. The evaluation results were presented in a table, but not using an overall score, as this gives equal importance to each psychometric property, which is not necessarily appropriate [14].

The cross-cultural adaptation process of the instruments was evaluated according to the five steps [15], namely: (1) translation, (2) back-translation, (3) committee review, (4) pre-test, and (5) re-examination of score weighting. In the first step, at least two qualified translators translated the scale from the original language into the target language. In the second step, two independent translators must translate the translated version back into the original language to ensure that the translation reflects the content of the original. The third step ideally involves a committee review to develop the penultimate version for pre-testing, and the fourth step consists of applying this version among 30–40 individuals from the target population. The final step is to re-examine the weighting of scores considering the cultural context.

### Risk of bias assessment

The risk of bias was evaluated using the COSMIN Risk of Bias Checklist [16]. This checklist includes three parts with 10 boxes. Boxes 1 and 7 to 10 were not applicable to this systematic review. Measurement properties related to content validity (box 2), internal structure (boxes 3 to 5), and cross-cultural validity (box 6) were assessed. Each included article was assessed using “very good,” “adequate,” “doubtful,” and “inadequate” to grade the above five domains. Two reviewers (DWDdeO and FSL) independently completed this assessment of the included study, with discrepancies solved through consensus.

### Certainty assessment

The certainty of evidence was assessed according to the GRADE methodology using the GRADEpro program, depending on each analyzed outcome (psychometric properties and cross-cultural adaptation). It was classified as high, moderate, low, or very low. The starting point always assumes that the pooled or overall result is of high quality. The certainty of evidence was reduced by one or two levels when risks of bias, inconsistency, imprecision, and/or indirectness were identified.

## Results

### Search and selection

**Fig. 1:**
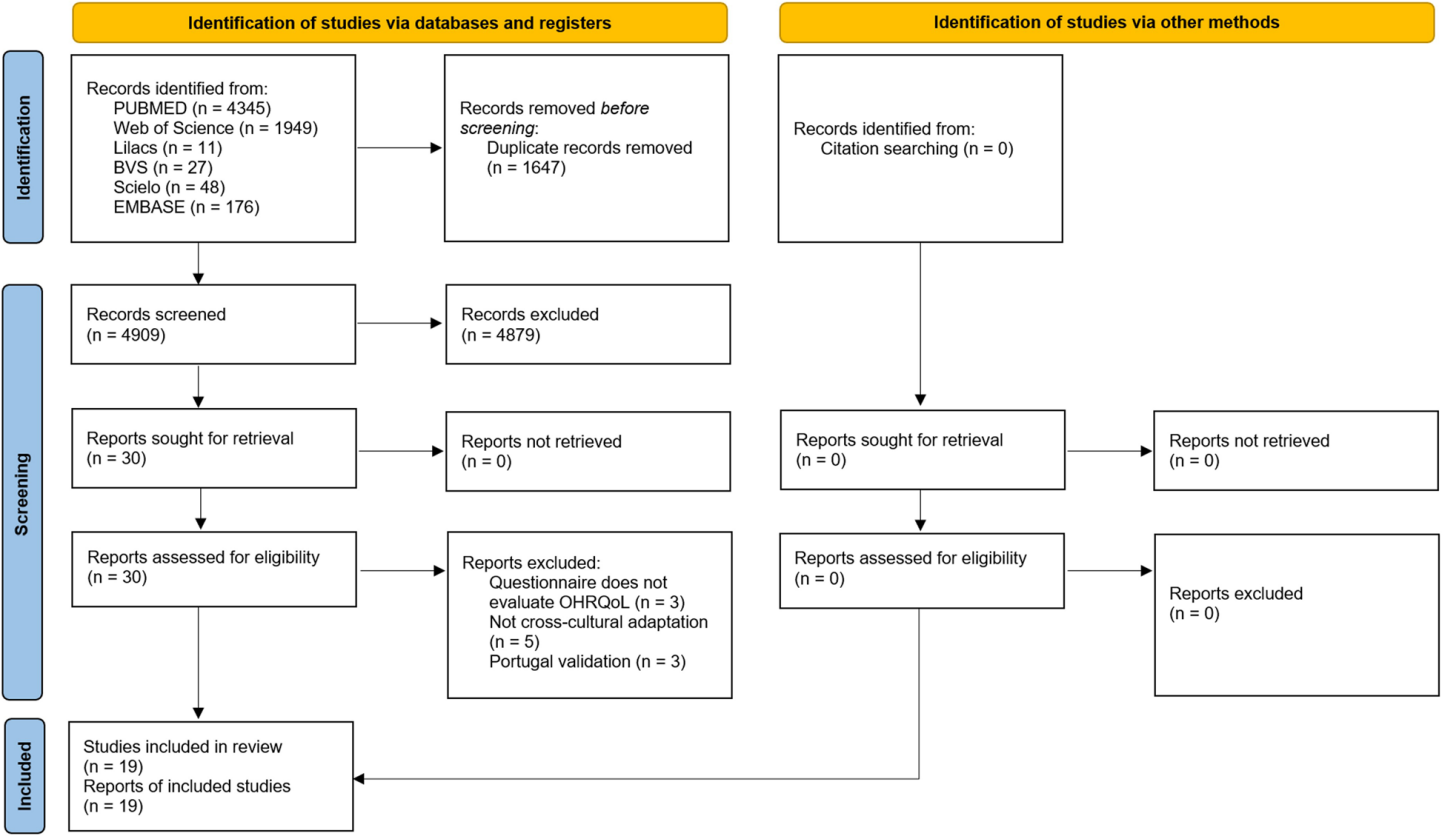
Flowchart of the included studies

A total of 6556 articles were identified in the databases, and 1647 duplicates were removed. The manual search did not identify additional studies. In the first phase, 4879 publications were excluded. In the second phase, 11 studies were excluded (Supplement 1). Therefore, 19 articles were included in this review [17–35] (Fig. 1).

### Qualitative assessment

All studies [17–35] have a cross-sectional design and were carried out in Brazil. The mean age of participants ranged from 2 [20] to 15.42 years [22], however, two studies did not report this information [21, 32]. The number of participants ranged from 20 [32, 35] to 342 [25]. Three studies did not go through the cross-cultural adaptation process [17, 28, 29] (Table 2).

**Table 2:** Characteristics of the reviewed studies

Study	Country	Study design	Participants	Original language	Cross-cultural adaptation	Psychometric validation	Target population	Main results reported	Conclusion
**PAIVA et al.,2018 **[18]	Brazil	Cross-sectional	55 men and 36 women, 14.7 years-old	English	Present	Present	Children with cancer	Regarding internal consistency, Cronbach's alpha (a) values were 0.769 (95% CI ¼ 0.631e0.868) and 0.879 (95% CI ¼ 0.872e0.920) for the self-reported and proxy versions, respectively. The convergent validity criteria were met for the self-reported and proxy versions (Spearman's rho ¼ 0.466e0.751; *P* < 0.001 and Spearman's rho ¼ 0.410e0.551; *P* < 0.001, respectively). The test-retest reliability assessment for the total score and items 1, 2, 3, and 4 in both versions showed a $0.7 correlation coefficient	The self-reported and proxy versions in Portuguese of the ChIMES were considered culturally adapted, valid, and reliable for Brazilian pediatric patients aged between one month and 18 years and were named ChIMES-BR.
**REBOUÇAS et al.,2018 **[23]	Brazil	Cross-sectional	70 boys and 91 girls, 13.84 years old	English	Present	Present	Adolescent using fixed orthodontic appliances	The B-IFAM overall score showed high correlation coefficients with most subscales (r = 0.52–0.74), supporting construct validity. The validity discriminant showed a statistically significant difference in subscales of overall score, aesthetics, and physical impact among children/female and male adolescents (*p* < 0.05)	The overall B-IFAM score and some subscales demonstrated adequate psychometric properties regarding reliability and validity. The study achieved a condition-specific instrument feasible for use in Brazilian children/adolescents who use fixed orthodontic appliances.
**MARTINS et al.,2018 **[34]	Brazil	Cross-sectional	42 boys and 58 girls, 10.1 years old	English	Present	Present	Children	The results of the internal validity analysis indicated adequate internal consistency and statistically significant internal congruence in the two factors identified in factorial analysis.	The Brazilian version of the COHIP-SF 19 showed good internal consistency but lacked external validity when compared to CPQ11-14.
**SANTOS et al.,2016 **[28]	Brazil	Cross-sectional	90 boys and 104 girls, 13 years old	NR	Absent	Present	Adolescents	Discriminant validity revealed a significant difference between the mean scores for the domains of dental self-confidence and psychological impact between groups with and without malocclusion.	The Brazilian version of the PIDAQ for adolescents has satisfactory psychometric properties and applies to this age group in Brazil
**DAHER et al.,2014 **[21]	Brazil	Cross-sectional study	30 parents/ guardians of children, age N.R.	English	Present	Present	Children	Conceptual and item analyses showed that there are similarities in the DDQ construct between the original and Brazilian cultures that require small modifications. The translations and back-translations allowed the development of the preliminary version in Brazilian Portuguese of the DDQ, which was tested and has undergone other minor changes to improve its understanding.	A Brazilian-Portuguese version of the DDQ was presented.
**DAHER et al.,2014 **[21]	Brazil	Cross-sectional	154 boys and 109 girls, 43.5 months old.	English	Absent	Present	Children	The factorial exploratory analysis revealed an instrument with 3 domains. The instrument showed excellent stability.	The DDQ-B proved to be reliable and with good psychometric properties to assess this group of Brazilian children with toothache due to caries.
**ABANTO et al.,2013 **[26]	Brazil	Cross-sectional	106 boys and 87 girls, 5 to 6 years old.	English	Present	Present	Children	Construct validity was satisfactory and showed consistent and strong associations between SOHO-5 and different subjective global oral health classifications (*p* < 0.001). SOHO-5 was able to discriminate between children with and without a history of dental caries (*p* < 0.001).	The SOHO-5 has satisfactory psychometric properties and applies to children aged 5 to 6 years in Brazil.
**MARTINS-JUNIOR et al.,2012 **[17]	Brazil	Cross-sectional	123 boys and 124 girls, 48.5 months	English	Absent	Present	Preschool children	The child impact section (*p* <0.01), family impact section (*p* < 0.01), and total ECOHIS scores (*p* < 0.01) were significantly correlated with tooth decay. Cronbach’s alpha coefficients demonstrated satisfactory internal consistency.	The Brazilian version of the ECOHIS is a valid instrument for assessing oral health-related quality of life in preschool children with Brazilian Portuguese-speaking primary caregivers.
**BENDO et al.,2012 **[24]	Brazil	Cross-sectional	86 boys and 122 girls, 7,96 years old	English	Present	Present	Children and adolescents	Confirmatory factor analysis showed that the five items from the child's self-report and the parent's proxy report were loaded into a single construct. Cronbach's alpha coefficients for child/adolescent and parent instruments were 0.65 and 0.59. Test-retest reliability (ICC) for child/adolescent and parent were 0.90 and 0.86.	The feasibility, reliability and validity of the Brazilian version of the PedsQLTM Oral Health Scale for self-report of children aged 5 to 18 years and parental proxy report for children aged 2 to 18 years was confirmed.
**DE SOUZA BARBOSA et al.,2011**	Brazil	Cross-sectional	38 boys and 22 girls, 11 to 14 years old	English	Present	Absent	Children	The understanding of the questionnaire was low for children aged 11 to 14 years and the necessary adaptations were made. The Portuguese version was considered adequate for more than 95% of the children evaluated.	The Portuguese version of CPQ11-14 is a useful tool to assess the quality of life in Brazilian children.
**BARBOSA et al.,2011 **[30]	Brazil	Cross-sectional	20 subjects, 8 to 10 years old	English	Present	Absent	Children	Terms that were incompatible for the cultural context of the population analyzed was substituted. In the pre-test, the Brazilian version of CPQ8-10 was clearly understood by the population studied.	The Portuguese version of the CPQ8-10 proved to be fully comprehensible to the Brazilian child population
**PIMENTA et al.,2010 **[22]	Brazil	Cross-sectional	157 men and 47 women, 15.42 years-old	English	Present	Present	Adolescents	The internal consistency obtained was 0.52. Interobserver and intraobserver correlations were strong, 0.87 and 0.83, respectively. The correlation with the aesthetic part of the OIDP was 0.44.	The results showed that the cross-cultural adaptation process was successful, and the instrument adaptation presented good psychometric properties.
**BARBOSA et al.,2010 **[32]	Brazil	Cross-sectional	20 subjects, age NR	English	Present	Present	Children	The findings suggest the adequacy of the process of cultural adaptation of the instrument to the Portuguese language. In the pre-test, the questionnaire was presented with good understanding.	The version in Portuguese of the P-CPQ proved to be easy to understand by the population of Brazilian parents.
**MARTINS et al.,2009 **[19]	Brazil	Cross-sectional	259 children, 8 to 10 years	English	Present	Present	Children	Cronbach's alpha=0.92 for the total scale and 0.63-0.85 for the subscales. ICC=0.96 for the total scale and 0.79-0.95 for the subscales. Construct validity was demonstrated through significant correlations between global indicators and subscales. There were significant differences between the clinical groups in relation to the total scale, demonstrating validity discriminant (p=0.03)	The preliminary validation of the Brazilian version of the CPQ8-10 proved to be valid and reliable for use in Brazilian children.
**GOURSAND et al.,2009 **[27]	Brazil	Cross-sectional	70 children, 11.89 years old	English	Present	Present	Children	The test-retest reliability revealed satisfactory reproducibility (ICC=0.83). The construct validity was satisfactory. The P-CPQ score was able to discriminate between the different perceptions of parents/caregivers about their children's oral conditions (dental caries and malocclusion).	This questionnaire is a reliable and valid instrument to assess parents' perception of the impact that oral health status has on children's quality of life.
**TORRES et al.,2009 **[33]	Brazil	Cross-sectional	56 boys and 80 girls, 12.7 years old	English	Present	Present	Children	Construct validity was confirmed by the correlation between short-form scores and ratings of oral health and general well-being. The CPQ11-14 short-form scores were able to discriminate between different oral conditions. Criterion validity was satisfactory (*p* < 0.05).	The Brazilian versions of the CPQ11–14 ISF:8 and ISF:16 have satisfactory psychometric properties, similar to those of the original instrument.
**TESCH et al,.2008 **[20]	Brazil	Cross-sectional	20 parents/ guardians of children 2-5 years of age	English	Present	Absent	Children	The translated versions were very similar and after carrying out all the steps, a final version of the ECOHIS was obtained.	A Brazilian version of the ECOHIS was obtained, which has semantic equivalence with the original instrument.
**CASTRO et al., 2008 **[25]	Brazil	Cross-sectional	170 boys and 172 girls, 12.8 years old	English	Present	Present	Children	Cronbach's alpha was 0.63, weighted kappa 0.76, and intraclass correlation coefficient (ICC) 0.79. The index had a significant association with self-reported health measures (*p* < 0.01).	The Child-OIDP index is a measure of oral health and quality of life that can be applied to Brazilian children.
**GOURSAND et al.,2008 **[31]	Brazil	Cross-sectional	80 boys and 80 girls, 12 years old	English	Present	Present	Children	Internal reliability was confirmed by a Cronbach's alpha coefficient of 0.86. Test-retest reliability revealed satisfactory reproducibility (ICC = 0.85). Construct validity was satisfactory. The instrument was able to discriminate between different oral conditions (groups without and with untreated caries)	This study provides evidence supporting the cross-cultural validity of a Brazilian Portuguese version of CPQ11–14 that can be recommended as an OHRQoL measurement for Brazilian children from 11–14 years

Table 3 presents the health conditions assessed by the instruments. The self-completion method [18, 23, 27–33, 35], interviews answered by the participants themselves [19, 22, 24–26], and interviews answered by parents [17, 20, 21, 26] were used to fill out the questionnaires. The domains/dimensions of the questionnaires were not reported in the two studies [22, 26]. The Brazilian version of the cross-culturally validated instrument was available in only six publications [18, 20, 21, 25, 32, 34].

**Table 3:** Characteristics of oral health-related quality of life questionnaires

Study	Instrument	Abbreviation	Generic/specific instrument	Completion method	Domain names	Number of items	Score	Evaluation period	Completion time	Available instrument
**PAIVA et al.,2018 **[18]	Children’s International Mucositis Evaluation Scale	ChIMES	Specific condition: oral mucositis in children with cancer	Self-completion	Functional domain: pain, speech, swallowing, eating.	ChIMES (6 itens) OMDQd Versão Pediátrica ( 7 itens)	Items 1 to 4 are scored from 0 (best score) to 5 (worst score). The remainder of the items are answered with a yes or no response and are assigned scores of 1 and 0, respectively. The maximum total score is 23	NR	NR	Yes
**REBOUÇAS et al.,2018 **[23]	Impact of Fixed Appliances Measure	IFAM	Specific Condition: children and adolescents between 10 and 18 years old who use fixed orthodontic appliances	Self-completion (Answered by the patients)	Aesthetics; functional limitation; dietary impact; oral hygiene impact; maintenance impact; physical impact; social impact: time constraints; and travel/cost/inconvenience implications	43	The total ranges from 43 to 215 and a higher score denotes a greater negative impact of fixed orthodontic appliances on the daily lives of children and adolescents.	NR	NR	No
**MARTINS et al.,2018 **[17]	Child Oral Health Impact Profile	COHIP-SF19	Specific condition: children from 8 to 15 years old	Self-completion (Answered by the participants)	Self-perception of oral health, functional well-being, and social/emotional well-being.	19	NR	NR	15 minutes	Yes
**SANTOS et al.,2016 **[28]	Psychosocial Impact of Aesthetics Questionnaire	PIDAQ	Specific condition: presence of malocclusion	Self-completion (Answered by the participants)	Aesthetic concern, psychological impact, social impact and dental self-confidence	23	A higher score denotes a greater impact on the children’s quality of life	NR	NR	No
**DAHER et al.,2014 **[21]	Dental Discomfort Questionnaire	DDQ	Specific condition: Cchildren with toothache	Interview (Answered by the parents)	Pain, functional limitation, oral impact of daily activities.	12	NR	NR	NR.	Yes
**DAHER et al.,2014 **[29]	Dental Discomfort Questionnaire	DDQ	Specific condition: children with toothache	Self-completion (Answered by the parents)	Problems eating and sleeping, earache problems, problems brushing teeth.	12	NR	NR	NR	No
**ABANTO et al.,2013 **[26]	scale of oral health outcomes for 5-year-old children	SOHO-5	Generic instrument	Interview (Answered by the participants and the parents)	NR	7	A higher score denotes a greater degree of oral impact on the children's quality of life	NR	NR	No
**MARTINS-JUNIOR et al.,2012 **[17]	Early Childhood Oral Health Impact Scale	ECOHIS	Specific condition: caries	Interview (The original version of ECOHIS was developed to be a self-completed questionnaire. Due to the low level of education of most Brazilians, interviews were chosen. (answered by the parents).	Child symptoms, child function, child psychology and child self-image/social interaction, parental distress, and family function	13	A total score ranging from zero to 52 is calculated as a simple sum of the responses with higher scores denoting a greater oral health impact and/or poorer OHRQoL.	NR	NR	No
**BENDO et al.,2012 **[24]	Oral Health Scale Pediatric Quality of Life Inventory	PedsQLTM	Generic instrument	Interview (Answered by the participants)	Physical Functioning, Emotional Functioning, Social Functioning and School Functioning. (Physical, social, emotional, and functional impact)	23	Items are reverse-scored and linearly transformed to a 0 – 100 scale, so that higher scores indicate better OHRQoL	NR	NR	No
** BARBOSA et al.,2011 **[30]	Child Perceptions Questionnaire	CPQ11-14	Specific condition: children from 11 to 14 years old	Self-completion (Answered by the participants)	Oral symptoms, functional limitations, emotional well-being, and social well-being.	37	The final score can vary from 0 to 148, for which a higher score denotes a greater degree of the impact of oral conditions on the quality of life of the respondents	Last 3 months	NR	No
**BARBOSA et al.,2011 **[35]	Child Perceptions Questionnaire 8-10	CPQ 8-10	Specific condition: children from 8 to 10 years old	Self-completion (Answered by the participants)	Oral symptoms, functional limitations, emotional well-being	29	The higher the score, the greater the impact on the quality of life	NR	NR	No
**PIMENTA et al.,2010 **[22]	Oral Aesthetic Subjective Impact Score	OASIS	Specific condition: oral aesthetics	Interview (Answered by the adolescents)	NR	5	The value of OASIS varies from 5 to 35 points. The higher the final value, the more likely a greater negative perception of oral aesthetics	NR	NR	No
**BARBOSA et al.,2010 **[32]	Parental-Caregiver Perceptions Questionnaire	P-CPQ	Generic instrument	Self-completion (Answered by the parents)	Oral symptoms, functional problems, emotional well-being, social well-being	35	The higher the score, the greater the impact of oral diseases on quality of life	NR	NR	Yes
**MARTINS et al.,2009 **[19]	Child Perceptions	CPQ8-10	Generic instrument	Interview (Answered by the children)	Oral symptoms, functional limitations, emotional well-being, and social well-being	25	The total score ranges from 0 (no impact of oral condition on quality of life) to 100 (maximum impact of oral condition on quality of life).	Last 4 weeks	NR	No
**GOURSAND et al.,2009 **[27]	Parental-Caregiver Perceptions Questionnaire	P-CPQ	Generic instrument	Self-completion (Answered by the parents)	Oral symptoms, functional limitations, emotional well-being, and social well-being	33	NR	NR	NR	No
**TORRES et al.,2009 **[33]	Child Perceptions Questionnaire (short forms)	CPQ11–14	Specific condition: children from 11 to 14 years old	Self-completion (Answered by the participants)	Oral symptoms, functional limitations, emotional well-being, and social well-being.	8 (ISF-8) and 16 (ISF-16)	As there are 16 and 8 questions, the final scores range from 0 to 64 and 0 to 32, for which a higher score denotes a greater degree of the impact of oral conditions on the quality of life.	NR	NR	No
**TESCH et al,.2008 [20[**	Early Childhood Oral Health	ECOHIS	Generic instrument	Interview (Answered by the parents)	Impact of oral problems on the child (child subscale) and impact of oral problems on the child's family	13	NR	The child's whole life from birth	NR	Yes
**CASTRO et al., 2008 [2005]**	Child Oral Impacts on Daily Performances	Child-OIDP	Generic instrument	Interview (Answered by the participants)	Functional, psychological, and social dimensions	8	The final Child-OIDP score ranges from 0 to 100.	Last 3 months	NR	Yes
**GOURSAND et al.,2008 **[31]	Child Perceptions Questionnaire	CPQ11–14	Specific condition: children from 11 to 14 years old	Self-completion (Answered by the participants)	Oral symptoms, functional limitations, emotional well-being, and social well-being.	37	The final score can vary from 0 to 148, for which a higher score denotes a greater degree of the impact of oral conditions on the quality of life of the respondents	NR	NR	No

*NR* Not reported

### Measurement properties and risk of bias assessment

The psychometric evaluation process, internal consistency, criterion validity, construct validity, reliability, general discriminant validity, Cronbach's alpha value, and general ICC value are presented in Table 4. The stages of the cross-cultural adaptation process; translation, back-translation, committee approach, synthesis, and pre-test were absent in three studies [17, 21, 28] (Table 5).

**Table 4:** Evaluation of the psychometric properties

Study	Content Validity	Internal Consistency	Criterion validity	Construct validity	Reliability	Discriminant validity	Cronbach's global alpha value	Overall ICC value
**PAIVA et al.,2018 **[18]	+	+	+	-	+	-	0.76	0.81
**REBOUÇAS et al.,2018 **[23]	+	+	+	+	+	+	0.89	0.81
**MARTINS et al.,2018 **[34]	+	+	+	+	+	-	0.68	0.65
**SANTOS et al.,2016 **[28]	-	+	-	+	+	+	0.59-0.86	0.54-0.89
**DAHER et al.,2014 **[21]	+	-	-	-	-	-	NR	NR
**DAHER et al.,2014 **[29]	-	+	+	+	+	+	0.75-0.81	0.74-0.97
**ABANTO et al.,2013 **[26]	+	+	+	+	+	+	0.77	0.92
**MARTINS-JUNIOR et al.,2012 **[17]	-	+	+	+	+	+	0.86	0.94
**BENDO et al.,2012 **[24]	+	+	+	+	+	+	0.85	0.90
**BARBOSA et al.,2011 **[30]	+	-	-	-	-	-	NR	NR
**BARBOSA et al.,2011 **[35]	+	-	-	-	-	-	NR	NR
**PIMENTA et al.,2010 **[22]	+	+	+	+	+	-	0.52	0.83
**BARBOSA et al.,2010 **[32]	+	-	-	-	-	-	NR	NR
**MARTINS et al.,2009 **[19]	+	+	+	+	+	+	0.92	0.96
**GOURSAND et al.,2009 **[27]	+	+	+	+	+	+	0.84	0.83
**TORRES et al.,2009 **[33]	+	+	+	+	+	+	0.70-0.84	0.98-0.97
**TESCH et al,.2008 **[20]	+	-	-	-	-	-	NR	NR
**CASTRO et al., 2008 **[25]	+	+	+	+	+	-	0.63	0.79
**GOURSAND et al.,2008 **[31]	+	+	+	+	+	+	0.86	0.85

*NR* Not reported

**Table 5:** Evaluation of the cross-cultural adaptation process

Study	Translation	Back translation	Synthesis	Committee's Approach	Pre-test	Psychometric Evaluation
**PAIVA et al.,2018 **[18]	Present	Present	Present	Present	Present	Present
**REBOUÇAS et al.,2018 **[23]	Present	Present	Present	Present	Present	Present
**MARTINS et al.,2018 **[34]	Present	Present	Present	Present	Present	Present
**SANTOS et al.,2016 **[28]	Absent	Absent	Absent	Absent	Absent	Present
**DAHER et al.,2014 **[21]	Present	Present	Present	Present	Present	Absent
**DAHER et al.,2014 **[29]	Absent	Absent	Absent	Absent	Absent	Present
**ABANTO et al.,2013 **[26]	Present	Present	Present	Present	Present	Present
**MARTINS-JUNIOR et al.,2012 **[17]	Absent	Absent	Absent	Absent	Absent	Present
**BENDO et al.,2012 **[24]	Present	Present	Present	Present	Present	Present
**BARBOSA et al.,2011 **[30]	Present	Present	Present	Present	Present	Absent
**BARBOSA et al.,2011 **[35]	Present	Present	Present	Present	Present	Absent
**PIMENTA et al.,2010 **[22]	Present	Present	Present	Present	Present	Present
**BARBOSA et al.,2010 **[32]	Present	Present	Present	Present	Present	Absent
**MARTINS et al.,2009 **[19]	Present	Present	Present	Present	Present	Present
**GOURSAND et al.,2009 **[27]	Present	Present	Present	Present	Present	Present
**TORRES et al.,2009 **[33]	Present	Present	Present	Present	Present	Present
**TESCH et al,.2008 **[20]	Present	Present	Present	Present	Present	Absent
**CASTRO et al., 2008 **[25]	Present	Present	Present	Present	Present	Present
**GOURSAND et al.,2008 **[31]	Present	Present	Present	Present	Present	Present

 The results of the risk of bias assessment are presented in Table 6. All studies were rated very good in the structural validity domain.

**Table 6:** COSMIN risk of bias assessment

Study	Box 2 (content validity)	Box 3 (structural validity)	Box 4 (internal consistency)	Box 5 (Cross-cultural validity)	Box 6 (Reliability)
**PAIVA et al.,2018 **[18]	Very good	Very good	Very good	Very good	Very good
**REBOUÇAS et al.,2018 **[23]	Very good	Very good	Very good	Very good	Very good
**MARTINS et al.,2018 **[34]	Very good	Very good	Very good	Very good	Very good
**SANTOS et al.,2016 **[28]	Inadequate	Very good	Very good	Inadequate	Very good
**DAHER et al.,2014 **[21]	Very good	Very good	Inadequate	Very good	Inadequate
**DAHER et al.,2014 **[29]	Inadequate	Very good	Very good	Inadequate	Very good
**ABANTO et al.,2013 **[26]	Very good	Very good	Very good	Very good	Very good
**MARINS-JUNIOR et al.,2012 **[17]	Inadequate	Very good	Very good	Inadequate	Very good
**BENDO et al.,2012 **[24]	Very good	Very good	Very good	Very good	Very good
**BARBOSA et al.,2011 **[30]	Very good	Very good	Inadequate	Very good	Inadequate
**BARBOSA et al.,2011 **[35]	Very good	Very good	Inadequate	Very good	Inadequate
**PIMENTA et al.,2010 **[22]	Very good	Very good	Very good	Very good	Very good
**BARBOSA et al.,2010 **[32]	Very good	Very good	Inadequate	Very good	Inadequate
**MARTINS et al.,2009 **[19]	Very good	Very good	Very good	Very good	Very good
**GOURSAND et al.,2009 **[27]	Very good	Very good	Very good	Very good	Very good
**TORRES et al.,2009 **[33]	Very good	Very good	Very good	Very good	Very good
**TESCH et al,.2008 **[20]	Very good	Very good	Inadequate	Very good	Inadequate
**CASTRO et al., 2008 **[25]	Very good	Very good	Very good	Very good	Very good
**GOURSAND et al.,2008 **[31]	Very good	Very good	Very good	Very good	Very good

### Certainty assessment

The certainty of the evidence was downgraded one level by risk of bias, and it was considered moderate for both psychometrics and adaptation outcomes (Table 7).

**Table 7:** Systematic review level assessment

Certainty assessment							Certainty
Number of studies	Study design	Risk of bias	Inconsistency	Indirectness	Imprecision	Other considerations	
**Psychometric Analysis**							
19	Observational studies	serious^a^	Not serious	Not serious	Not serious	Very strong association	⨁⨁⨁◯ Moderate
**Cross-Cultural Adaptation**							
19	Observational studies	serious^b^	Not serious	Not serious	Not serious	Very strong association	⨁⨁⨁◯ Moderate

^**a**^Studies did not perform psychometric analysis. ^**b**^Studies did not perform the translation and back-translation process

## Discussion

The quality-of-life assessment is an important parameter in several areas of health, including oral health, which allows an analysis of the condition's impact on daily activities and the individual's personal life [36]. However, clinical evaluation alone cannot analyze the psychosocial effects of oral health status and general well-being [37]. In this sense, it is necessary to use OHRQoL questionnaires to correctly assess this individual, understanding their multidimensionality and recording subjectivity in a uniform and reproducible way [38]. Nineteen OHRQoL instruments have been cross-culturally adapted for Brazil and had the psychometrics validated, and all of them proved to be valid and ready for use in children and adolescents.

All instruments included in this review had English as the original language [17–35]. Cultural and linguistic sensitivity is a common issue associated with the use of these questionnaires in non-English-speaking and/or cross-cultural populations, as certain items may not be relevant to all population groups. Therefore, translation and cross-cultural adaptation of these instruments are necessary when using them in a new country, culture, and/or language [39]. The reviewed studies were carried out in Brazil. They were all designed following the literature recommendations, which propose the use of cross-sectional studies in which data are collected in a single moment, without longitudinal follow-up. Studies using a cross-sectional design are very useful in several areas of research, especially in assessing the prevalence of diseases, attitudes, and knowledge among patients and health professionals [40]. Furthermore, this design is also used in validation studies comparing different measurement instruments and in reliability research [41].

Global population growth and the demand for cross-cultural studies highlight the importance of having reliable and validated instruments or measures available to clinicians and researchers in diverse cultures and/or languages [42]. However, among the reviewed studies, a few one provided the instruments adapted for Brazil [18, 20, 21, 25, 32, 34]. This situation can restrict the use of these instruments, limit the reference to the original studies, and even encourage other authors to develop similar instruments.

The average age of the participants ranged from 2 [20] to 15.42 years old [22]. Age is an important factor to be considered when evaluating the results reported by patients in childhood, as it influences not only the sources of information available but also the way they perceive and experience the quality of life-related to oral health. For this reason, it is crucial to develop specific assessment instruments for each age group [11].

The reviewed instruments were developed to be answered by the children themselves [18, 19, 22–26, 28, 30, 31, 33–35] or by their guardians [17, 20, 21, 27, 32] which is confirmed by the face validation. Quality of life assessment instruments for children should be segmented by different age groups, such as 6 to 7, 8 to 10, and 11 to 12 years old, and should be self-administered by the children themselves, since they have the right to voice their opinions and have their perspectives considered [10]. However, some groups of children, such as the very young ones, may have difficulty providing accurate information about their quality of life. For this reason, it is common for questionnaires aimed at preschoolers to be answered by their guardians [43, 44]. Adults and children have different perceptions about how health problems affect the quality of life, especially since children and adolescents have different views of themselves and the world given their physical and emotional development stages. Therefore, the development of specific instruments for children allows for a more accurate measurement of the impact of oral problems on their quality of life [10].

In the present review, both specific [17, 18, 21–23, 28, 30, 31, 33–35] and generic [19–21, 24–27, 32] instruments were identified, offering a broad range of options for researchers to choose from based on the study’s objective. Generic instruments are developed to represent the impact of a health condition on an individual's life and can be used in different populations [43]. They allow for assessing overall health and measures that demonstrate the patient's preference for a particular health state, treatment, or intervention [45]. In addition, they play an important role in allowing comparisons of health-related quality of life between patients who have different chronic diseases or even to assess the ORQoL of a single population concerning a disease; however, they are not able to detect situations experienced by patients with specific diseases [46].

On the other hand, specific instruments can individually assess specific aspects of quality of life, allowing a greater ability to detect positive or negative aspects. The main advantage of these instruments is their sensitivity to measure changes resulting from the natural history of the disease or after a specific intervention [47]. Some authors suggest that OHRQoL instruments aimed at specific conditions tend to be more sensitive to changes when compared to generic instruments, which have the advantage of being comprehensive and meeting all conditions and interventions [43]. This view is based on a focus on health aspects that are relevant to a specific group of patients, as evidenced by the inclusion of several items in each domain. However, the application of these specific instruments to different populations may make it impossible to compare these experiences. Consequently, it is common for the researcher to seek a combination of generic and specific instruments to obtain the desired response capacity and enable comparison between different groups [48].

The availability of these instruments to the researcher offers an enhanced opportunity for expression, language understanding, and evaluation, which develop into a more effective investigation and, therefore, promote the humanization of care [49]. Three studies did not undergo the process of cross-cultural adaptation [17, 21, 28]. The importance of these instruments going through the process of cross-cultural adaptation lies in their equivalence in different cultures, ensuring the preservation of their content, psychometric properties, and validity in a different cultural context [50]. Therefore, a flawed translation and adaptation process can result in unreliability, generating an inconsistency between the translated and original versions, which can compromise its validity and psychometric properties, affecting the reliability of a specific item or scale level [38].

Assessing the reliability of the data provided by these research instruments is critical and requires high-quality testing. In this sense, researchers must estimate this quantity to improve the validity and accuracy of the interpretation of their data [51]. The Alpha test is an important concept in the assessments of these questionnaires, as it measures the reliability and correlation between answers reported by patients [52]. An Alpha value greater than 0.70 is considered adequate for comparison between groups, indicating satisfactory internal consistency and the presence of a high Alpha coefficient (> 0.90) may imply the existence of redundancies [51].

The methods used in the evaluated studies to record the reports of individuals were the self-completion method (self-report scale) and the interview (evaluation scale). A good way to assess the child's subjective experience is through self-reports, which are accessible and easy to administer. With proper guidance, children can adequately describe the characteristics and levels of discomfort they are experiencing [53].

Reporting the time taken to complete these questionnaires is highly relevant information since the researcher would have prior knowledge about the time required for data collection when using the instrument. In this review, this information was mentioned in the study by [34]. Another important piece of data that should be considered in these instruments so that there is no response bias and/or methodological bias compromising the results found is the indication of the period to be considered in the participant's response [54, 55], information that was absent in most of the studies [17, 21–24, 26–28, 31–35].

GRADE is a tool used to assess the certainty of evidence in systematic reviews [56]. Moderate certainty of evidence suggests that the available data from the psychometric validation studies are generally reliable and provide a reasonable level of confidence in the findings. In other words, the results are likely to be accurate, but some uncertainty or limitations may still exist [13, 56]. These limitations could be due to potential bias in the study design caused by the absence of a translation process and psychometric validation. Researchers and practitioners should consider the limitations and uncertainties associated with the evidence when making decisions or drawing conclusions based on these instruments.

This review has some limitations, such as the lack of complete reports on the information investigated in some studies, the lack of publication of transcultural adapted instruments, and the lack of analysis of the longitudinal validation of the reviewed studies. In this sense, cross-sectional studies are recommended to validate the oral health-related quality of life instruments adapted for the Brazilian context. It is suggested that researchers publish the OHRQoL instruments that have already been validated, in addition to using the guidelines proposed in the literature to ensure equivalence of content with the original scale.

## Conclusion

It can be concluded that most studies provided information and evidence regarding validity, reliability, translation, and cultural adaptation. The quality of the evidence was moderate, and five papers failed to establish the reliability of PIDAQ, DDQ-B, ECOHIS, CPQ8-10, and CPQ11-14 Brazilian version instruments. Overall, the oral health-related quality of life questionnaires adapted for children and adolescents were considered valid for use in Brazil.

### Supplementary Information

**Additional file 1: Supplement 1.** List of the excluded articles with reasons.

**Additional file 2.** PRISMA 2020 Checklist.

## Data Availability

The datasets used and/or analyzed during the current study are available from the corresponding author upon reasonable request.
