# Supplementary material for: Reliability and validity of cross‑culturally adapted oral health‑related quality‑of‑Life instruments for Brazilian children and adolescents: a systematic review
Source: BMC Oral Health. 2024 Feb 10;24:214. doi: 10.1186/s12903-024-03940-4 (PMC10859033; doi:10.1186/s12903-024-03940-4)
Supplement: Supplementary file 1 — Additional file 1: Supplement 1. List of the excluded articles with reasons. [file 12903_2024_3940_MOESM1_ESM.docx]

| **Supplement 1.** List of the excluded articles with reasons. | |
| --- | --- |
| **Article** | **Exclusion reason** |
| Soares RC, Moysés ST, Rocha JS, Baldani MH, Werneck RI, Moysés SJ. Cross-cultural adaptation of the Dental Neglect Scale for five-year-old children in Brazil. Braz Oral Res. 2021;35:e115. | Questionnaire does not evaluate oral health-related quality of life. |
| Silva SB da, Cabral T de M, Pinto TMP, Durand LB. Instrument of self-perception and knowledge of dental erosion: cross-cultural adaptation to the Brazilian population. Braz Oral Res. 2015;29(1):53. | Questionnaire does not evaluate oral health-related quality of life. |
| Hilasaca-Mamani M, Barbosa T de S, Fegadolli C, Castelo PM. Validity and reliability of the quality of masticatory function questionnaire applied in Brazilian adolescents. CoDAS . 2016;28(2):149–54. | Questionnaire does not evaluate oral health-related quality of life. |
| Campos JADB, Carrascosa AC, Zucoloto ML, Maroco J. Validation of a measuring instrument for the perception of oral health in women. Braz Oral Res [Internet]. 2014;28(1):1–7. | Not cross-cultural adaptation. |
| Mello dos Santos C, Hugo FN, Leal AF, Hilgert JB. Comparison of two assessment instruments of quality of life in older adults. Rev Bras Epidemiol [Internet]. 2013;16(2):328–37. | Not cross-cultural adaptation. |
| Possebon AP da R, Faot F, Machado RMM, Nascimento GG, Leite FRM. Exploratory and confirmatory factorial analysis of the OHIP-Edent instrument. Braz Oral Res . 2018;32(0):e111. | Not cross-cultural adaptation. |
| Abanto J, Tsakos G, Ardenghi TM, Paiva SM, Raggio DP, Sheiham A, et al. Responsiveness to change for the Brazilian Scale of Oral Health Outcomes for 5-year-old children (SOHO-5). Health Qual Life Outcomes. 2013;11(1):137. | Not cross-cultural adaptation. |
| Abanto J, Paiva SM, Sheiham A, Tsakos G, Mendes FM, Cordeschi T, et al. Changes in preschool children’s OHRQoL after treatment of dental caries: responsiveness of the B-ECOHIS. Int J Paediatr Dent. 2016;26(4):259–65. | Not cross-cultural adaptation. |
| Godinho GF,Cavalheiro A, Luís HS,Mexia R. Validation of the Oral Impacts on Daily Performance index among the Portuguese population.Ciência & Saúde Coletiva.2018;23 (12):4351-4361. | Portuguese from Portugal. |
| Amaral J, Sanches C, Marques D, Vaz Patto J, Barcelos, F, Mata,A.Validation of oral health impact profile-14 and its association with hypossialia in a Sjögren's Syndrome Portuguese population. Acta Reumatologica Portuguesa , 2018;43(2). | Portuguese from Portugal. |
| Da Mata ADSP, da Silva Marques DN, Freitas FMF, de Almeida Rato Amaral JP, Trindade RTVMR, Barcelos FAD, et al. Translation, validation, and construct reliability of a Portuguese version of the Xerostomia Inventory: Portuguese version of the Xerostomia Inventory. Oral Dis. 2012;18(3):293–8. | Portuguese from Portugal. |
